# Moisturizing effects of solid lipid nanoparticles (SLN) and nanostructured lipid carriers (NLC) using deionized and magnetized water by *in vivo* and *in vitro* methods

**DOI:** 10.22038/IJBMS.2020.39587.9397

**Published:** 2020-03

**Authors:** Susan Sarhadi, Mostafa Gholizadeh, Tina Moghadasian, Shiva Golmohammadzadeh

**Affiliations:** 1 Department of Pharmaceutical Nanotechnology, School of Pharmacy, Mashhad University of Medical Sciences, Mashhad, Iran; 2 Department of Chemistry, Faculty of Science, Ferdowsi University of Mashhad, Mashhad, Iran; 3 Nanotechnology Research Center, Pharmaceutical Technology Institute, Mashhad University of Medical Sciences, Mashhad, Iran

**Keywords:** Deionized water, Magnetized water, NLC, Skin dryness, SLN

## Abstract

**Objective(s):**

The present study aimed to determine and compare moisturizing and occlusion effects of different solid lipid nanoparticles (SLN) and nanostructured lipid carriers (NLC) using magnetized water and deionized water.

**Materials and Methods:**

SLN formulations were prepared using various lipids, including Tripalmitin, Compritol®, Precirol®, and emulsifiers including Poloxamer and Tween 80. NLC formulations were also prepared with oleic acid and the same solid lipids. Two types of formulations were prepared; first with deionized water and then with magnetized water. Formulations were prepared using high shear homogenization and ultrasound methods. The products were analyzed by PSA (particle size analyzer), DSC (differential scanning calorimetry), and TEM (transmission electron microscopy). The moisturizing effect of formulations was determined by *in vivo* and *in vitro* methods.

**Results:**

Findings of the assessments demonstrated that in products prepared with magnetized water, 5% SLN Precirol® had the most moisturizing effect* in vivo* and 5% SLN Compritol® had the most moisturizing effect *in vitro*. The use of magnetized water in formulations can improve the effectiveness and increase the stability of moisturizing products.

**Conclusion:**

In this study, all products prepared with magnetized water showed more stability, smaller size, and more moisturizing effects compared with products prepared with deionized water.

## Introduction

Skin is a complex organ due to its various functions such as physical and chemical protection, vitamin D synthesis, gas exchange, protection against pathogens, as well as temperature regulation. It is also considered as a barrier to moisture loss from the body surface. Dry skin reduces the skin’s ability to perform its tasks. The function of a moisturizer is to restore or retain the moisture of the horny layer of the skin (1, 2).

In recent decades, solid lipid nanoparticles (SLN), made of biodegradable and biocompatible lipids, are locally used for a variety of reasons (3), some of which are as follows: 

1) Protecting unstable compounds from chemical degradation (3, 4)

2) Possibility of providing controlled release drugs (5, 6)

3) Moisturizing property due to their occlusive effect (2, 7) 

4) The possibility of increasing penetration and skin accumulation due to their occlusive properties (8)

5) Physical sunscreen due to UV-blocking (9)

Nanostructured lipid carriers (NLC) are a new generation of colloidal drug carriers for topical application. The goal of these carriers is to overcome the SLN problems. One of the differences between SLN and NLC is that SLN is composed only of solid lipids, while NLC is composed of a mixture of solid lipids and liquid lipids in a ratio of 70:30 up to a ratio of 99.9:0.1 (10).

The most important benefits of NLC include higher carrying capacity for some materials and less waste material during storage compared to SLN (11). SLN and NLC are among the novel skin drug delivery systems.

Various studies have shown that the occlusion factor of lipid microparticles is only 10%, while with a size of approximately 200 nm, the lipid nanoparticles increase this factor to 50% (12).

Studies show that when water is affected by the static magnetic field, some of its properties change, including reduced surface tension, increased viscosity, increased pH, and increased electrical conductivity and hydrogen bond (Table 1) (13). Moreover, the magnetic field can significantly affect the zeta potential and the dispersion of the particle size of solutions (14, 15). 

The present study aimed to determine and compare the moisturizing and occlusion effects of different SLN and NLC using magnetized water and deionized water. We developed SLN and NLC formulations using different solid and liquid lipids and various emulsifiers. All formulations were prepared with deionized water and magnetized water. The particle size of the nanoparticles was determined using PSA (particle size analyzer), the morphology of nanoparticles was studied using TEM (transmission electron microscopy); then, DSC (differential scanning calorimetry) analyses were performed. Finally, the occlusive factor and moisturizing effects were investigated using *in vivo* and *in vitro* methods.

## Materials and Methods

Glyceryl palmitostearate (Precirol ® ATO 5), Tripalmitin and Glyceryl behenate (Compritol® 888 ATO) were purchased from Gattefossé (Pvt. Ltd. France). Cetyl palmitate (CP) and Tween 80 were gifted from Sigma- Aldrich Co. (Deisenhofen Germany). Poloxamer 188 was obtained from Uniqema (Everberg, Belgium). Also, oleic acid was purchased from Oxin Chemistry Company (Kh. Razavi, Iran). The water used was either deionized or magnetized water. All of the materials were original and fresh. 

***Preparation of SLN and NLC***

SLN and NLC were prepared by high-shear homogenization and ultrasound technique. The lipid phase was heated at 5 ^°^C to 10 ^°^C above their melting point. In the current study, the lipid phase consists of Precirol®, Compritol®, and Tripalmitin. The aqueous phase was prepared by dissolving Tween 80 for Tripalmitin, Poloxamer for Compritol®, and Precirol® in deionized water and magnetized water (Table 2). Therefore, the hot aqueous phase was added to the melted lipid phase and then homogenized with a Diax 900 homogenizer (Heidolph, Germany) in three consecutive steps. The first step was 2.5 min at 18000 rpm, the second step for 2.5 min at 23000 rpm, and the third step for 1 min at 28000 rpm, homogenized. Throughout these steps, the temperature was kept up to 5 °C up to the lipid melting point. Then, the oil in water emulsion was ultrasonicated with a Probe Sonicator (Bransonic, USA) in 6 cycles of 30 sec with a rest period of 15 sec between each cycle. Finally, the mixture was placed at room temperature to cool completely, and the nanoemulsion was formed (16). The preparation of NLC was similar to SLN, except that it contained 30% oleic acid and 70% solid lipid. These formulations were prepared with both deionized water and magnetized water (17-20).

***Preparation of magnetized water***

**Figure 1:**
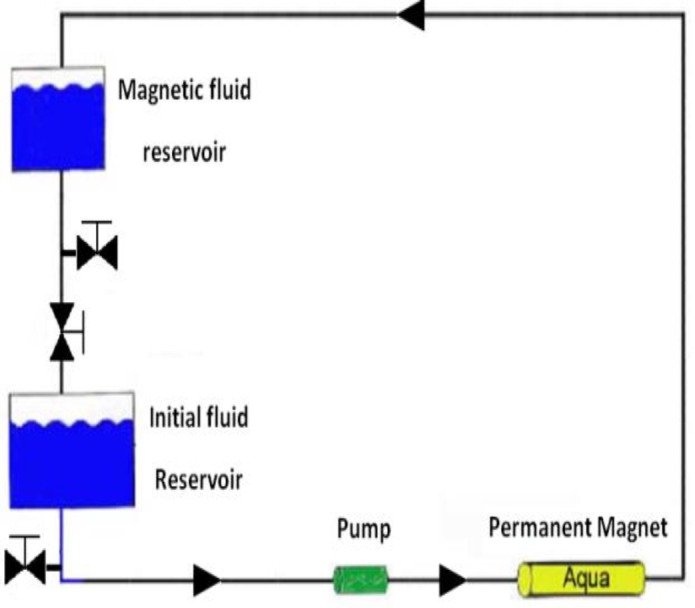
Schematic of a solvent magnetizing apparatus (SMA) with permission (21)

For this purpose, a solvent magnetizing apparatus (SMA) was used. The SMA had a static magnetic field in a compact form called “AQUA CORRECT.” The device (Germany, H.P.S Co.) had a coaxial static magnetic system of 6000 G field strength [DN = 20, 3/4 in., flow 2 m3/hr] for the experiments. It passes the water through the SMA, a magnetic source embedded in a hollow cylinder, and when it passes through this magnetic field, its properties are subject to change. First, the device was washed with deionized water carefully, so that about 1 liter of deionized water entered (1st lower reservoir of the apparatus) and then after closing the valve between the two reservoirs, the pump was turned on for 5 sec until all of the water passed through the magnetic device to the second reservoir (this magnetized the water for one pass). In the next step, to determine the optimum magnetic moment, the deionized water samples, were added to the device after opening the red valve the desired time of 5 min, 10 min, 15 min, 30 min, 45 min, 60 min, and 90 min for recycling (Figure 1). Finally, the sizes of SLN and NLC were analyzed at each time point by a particle size analyzer. According to the data, the water obtained from 60 min rotary water device was considered as the best-magnetized water for this study (21).

***Characterization of SLN and NLC***

*Particle size and zeta potential*

The mean particle size, the polydispersity index (PDI), and the zeta potential of the SLN and NLC formulations were assessed by a Dynamic Light Scattering method (ZetaSizer Nano-ZS; Malvern Instruments Ltd., United Kingdom). The experiments were carried out in triplicate at a temperature of 25±2 ^°^C and an angle of 90 ^⁰^ to the incident beam (22).

*Transmission electron microscopy (TEM)*

To evaluate the morphology of SLN and NLC characterization, the TEM assessment was used. SLN and NLC were diluted 12 times with water, and then 20 μl of the sample was coated on a carbon-coated copper grid. After that, 20 μl of uranyl acetate 2% in water was placed on SLN and NLC, 30 sec later, it was dried with paper filter. After drying, the sample was observed under an electron microscope (23, 24).

*Differential scanning calorimetry* *(DSC)*

The melting and crystallization properties of crystalline materials were investigated with the Mettler DSC 821 ͤ (Mettler Toledo, GieBen, Germany). The device has two positions for reference and sample in its aluminum pans. Samples were heated up from the temperature of 25 ^°^C to 165 ^°^C (5 ^°^C / min) under nitrogen (5 ml /min). Then, the melting points of SLN and NLC formulations and bulk lipids were compared (7).

*Occlusive properties assessment*

The occultation amounts of the samples were determined by the *in vitro* method. In this study, 39 numbers of 100 mL beakers of 100 ml each, were filled with 50 ml of water and covered with a sodium acetate filter (cut-off size: 4 to 7 µm). Then, the amount of 13.3 mg /cm² of each of the SLN and NLC samples werewas spread completely on the filter surface. All samples were incubated at 32 °C and 50- –55% relative hydration (RH) and 10% CO₂ for 48 hr. After 24 hr and 48 hr, the weights of beakers were recorded.

(Eq. 1)                    F=100((A-B)/A)

A: The amount of water loss without the sample (as references)

B: The amount of water loss with the sample

F: Occlusive factor percentage (18, 25).

*Skin hydration measurement using corneometer*

A corneometer (Courage, Khazaka, Cologne, Germany) was used to measure the moisture content of the skin after using different formulations of SLN and NLC; the test was performed on six volunteers with normal skin under normal environmental conditions in the age range of 20–30 years (16, 26). They were asked to refrain from using any product on the test site one week before the test, also during the study period they were not under direct sunlight or lamps. All measurements were performed on a specific area of the skin. Before the topical application (time zero), skin moisture content was measured. Then at 0.5, 1, 3, and 5 hr after application, the moisture content was measured at three different points. The results were obtained as an average of three reported data (27). All experimental protocols were approved by the Ethical Committee of Mashhad University of Medical Sciences (approval number: IR. mums. sp.1395.85)

***Data analysis***

Data were analyzed using the Prism software package. The results were compared based on one-way ANOVA and Turkey’s multiple comparison tests. *P*<0.05 was considered statistically significant.

## Results

Six SLN and six NLC formulations were prepared with high-shear homogenization and ultrasound method. The lipid and surfactant (type and concentration), type of water, and lipid/surfactant ratio were differential parameters for SLN and NLC preparations. 

The mean diameter (z-average), PDI, and zeta potential of different SLN and NLC formulations prepared with deionized water and magnetized water were measured by PSA (Table 3). 

Data were expressed as mean± SD (n=3); statistical significance difference with NLC-pp, NLC-pp MAG, SLN-tt, and SLN-tt MAG was observed (*P*<0.001). SLN and NLC with Precirol® and Tripalmitin lipids had a significantly smaller size than Compritol® lipid. 

**Figure 2:**
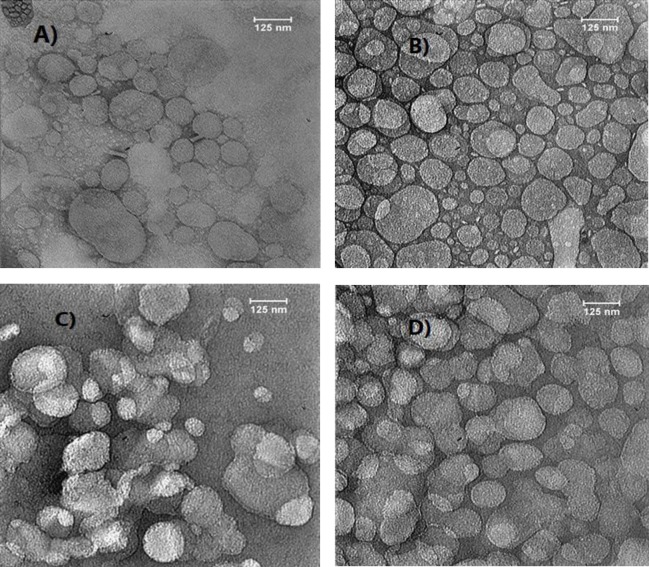
The transmission electron microscopy (TEM) images

The TEM imaging of SLN and NLC formulations with magnetized water was smaller, more spherical shaped, non-follicular, and more homogeneous (Figure 2). 

**Figure 3:**
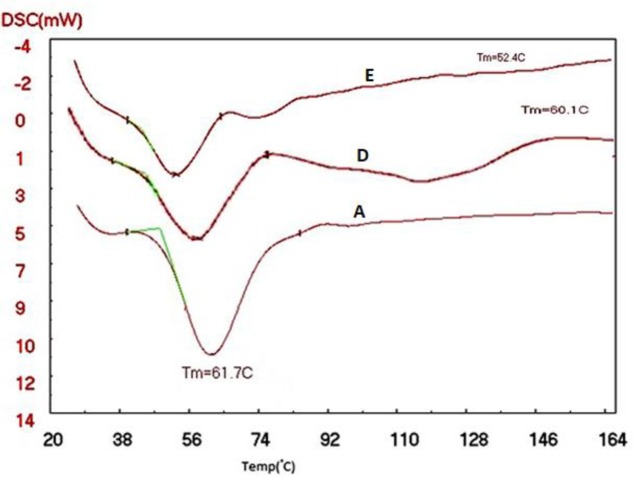
Differential scanning calorimetry (DSC) thermograms of (A)Precirol® bulk, D) SLN pp MAG: Precirol®, Poloxamer with magnetized water; E) SLN pp:Precirol®, Poloxamer with deionized water

**Figure 4:**
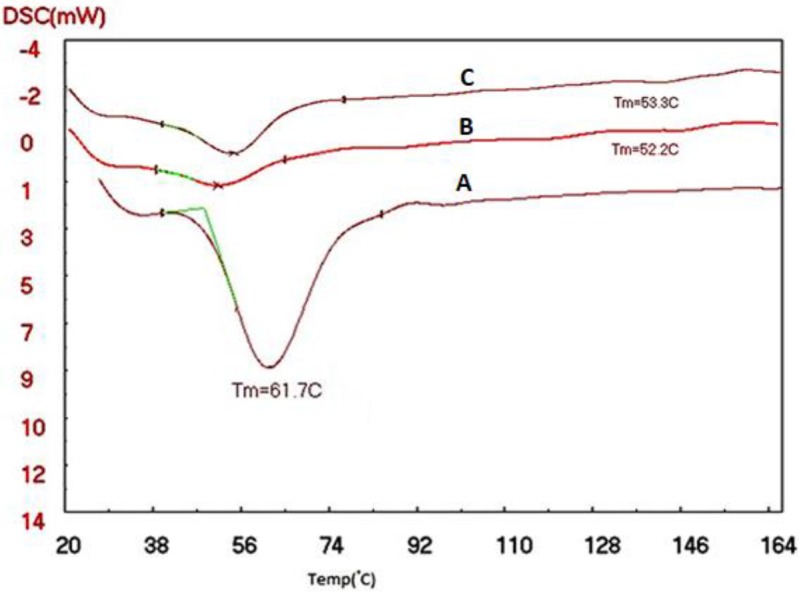
Differential scanning calorimetry (DSC) thermograms of (A)Precirol® bulk; B)NLC pp: Precirol®, Poloxamer with deionized water

DSC analyses of SLN and NLC were evaluated to determine the crystallinity of the formulations. SLN and NLC formulations with Precirol®, as the lipid, were scanned from 25 ^°^C to 165 ^°^C (5 ^°^C/min), and the melting points of the different formulations were compared with the bulk lipids. According to the diagram obtained from the DSC, the lipids peaked in NLC pp, NLC PP MAG, SLN pp, and SLN pp MAG slightly shifted towards lower temperatures compared to their bulk lipids (Figures 3 and 4). The recrystallization of the SLN and NLC formulations occurred at lower temperatures compared with the bulk material.

**Figure 5:**
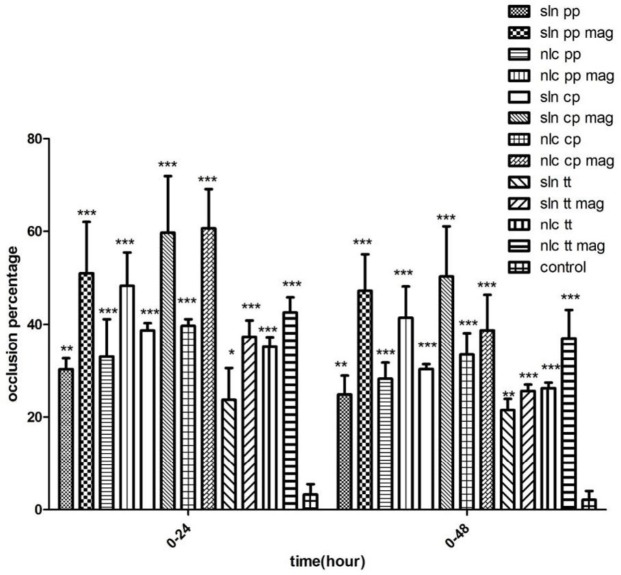
The occlusion factor (F) of different formulation of SLN and NLC after 24 hr and 48 hr (n=3)

After 24 hr, NLC formulations containing Compritol®, Precirol®, and Tripalmitin exhibited more occlusive properties than SLN formulations. The NLC containing the magnetized water and Comparitol® had the most occlusion factor ( Figure 5). 

After 48 hr of using the formulations, most occlusion factor was related to the SLN containing Compritol® and magnetized water. The SLN formulations prepared with magnetized water showed more occlusion factor (F) than the NLC formulations (except the SLN prepared with Tripalmitin). The NLC formulations containing Compritol®, Precirol®, Tripalmitin, and deionized water exhibited more occlusion properties than the SLN formulation (Figure 5).

***Skin hydration measurement using corneometer***

**Figure 6:**
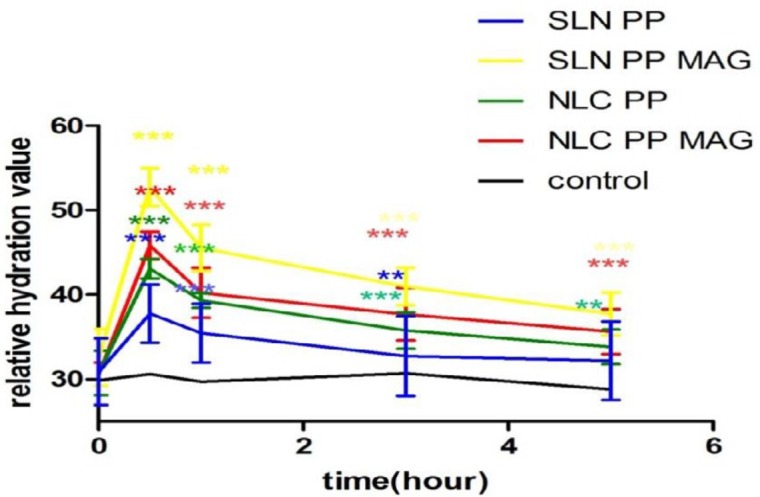
Normalized relative hydration values after application of different formulation of SLN and NLC compared to control without application of the product (n=6):(n=3 * *P*<0.05, ** P<0.01 and *** *P*<0.001). SLN pp: Precirol®, Poloxamer with deionized water; SLN pp MAG: Precirol®, Poloxamer with magnetized water; NLC pp: Precirol®, Poloxamer with deionized water; NLC pp MAG:Precirol®, Poloxamer with magnetized water

Figure 6 shows the moisturizing effect of 5% Precirol® formulations using a corneometer. The moisturization of each formulation has been evaluated on six volunteers and measured at different times (0.5, 1, 3, and 5 hr) compared with the control.

Compared to the control, SLN pp MAG and NLC pp MAG significantly increased the moisture content of the skin in all time points (*P*<0.001). At 1 hr application, the highest water content was observed for SLN pp MAG. After 5 hr, there was a significant difference between the samples of SLN Precirol® prepared with magnetized water and the control (Figure 6).

## Discussion

In this study, the diameter of the nanoparticles with magnetized water was significantly lower compared with the nanoparticles with deionized water. In formulations with magnetized water, the nucleation process took place more quickly but the growth process of the nuclei was slower; thus, the number of nanoparticles was increased, and each particle had a smaller size (28).

The important point of the stability of colloidal dispersions is the zeta potential. The high zeta potential (>30 mV) prevents the aggregation of the particles due to the presence of electric repulsion (29). The zeta potentials of SLN-cp MAG, NLC-cp MAG, and NLC-tt MAG were approximately -30 mV, showing more physical stability than other formulations. Various studies have shown that the magnetic field can affect zeta potential, since ion adsorption was changed (30, 31).

In the morphology evaluation, the nanoparticles containing magnetized water were better than other formulation. These characteristics are probably because of the reduction of hydrogen bonding in water molecules due to exposure to the magnetic field. When the water was exposed to the magnetic field, there was less hydrogen bonding with the functional groups of the used lipids; therefore, the lipids were not captured by water molecules, and the particles would be small, dispersed suitably, and not cumulated.

Reduction of melting temperature with DSC analysis in NLC pp, NLC PP MAG, SLN pp, and SLN pp MAG formulations can be attributed to several reasons: for example, the small particle sizes of the SLN and NLC formulations, their high specific surface area, as well as the presence of a surfactant. The DSC thermogram of NLC prepared with magnetized water shows a sharper pick than the NLC prepared with deionized water; because when the water is passing between two magnetic poles, it will get magnetized and break its molecular clusters into smaller parts. This enables the molecules to penetrate the reactant particles of NLC, as a result of the presence of water between the reactants, which will weaken their internal molecular bond between NLC. Consequently, we expect the crystalline network of the NLC to collapse with less heat .This can be due to the change in the liquid lipid and solid lipid particles placed in the NLC formulations, smaller particles, and more regular structure produced by magnetized water. 

In various studies it has been shown that occlusion factor is affected by particle size, volume, concentration of sample, lipid crystallinity, and type of colloidal systems (18). In the present study, all formulations prepared with magnetized water showed more occlusion properties than the formulations prepared with deionized water. The higher occlusion factor in magnetized water in SLN and NLC formulations is probably due to the smaller particle size, more regular lipid structure, greater penetration in cellulosic filter pores, and consequently, the increased blockage of the filter compared to deionized water. These reasons also showed more moisturizing effects in the *in vivo* method.

In a study, Souto *et al*. indicated that the amount of hydration of SLN is dependent upon the particle size, lipid concentration, and the crystallinity of lipid (18). Another study showed that SLN- CP (Cetyl palmitate) had more hydration than other formulations (SLN-GMS (glycerol monostearate) and SLN-P (Precirol®)), as the lipid crystallinity level of cetyl palmitate was more than the other lipids (16). In the current study, the highest moisture content at all time points (0.5, 1, 3, and 5 hr) was related to formulations prepared with magnetized water. When the lipid particles are applied onto the skin, a film layer will be formed on it; the surface area of this layer depends on the particle size. If the particle size is too small, a smaller air channel will be formed, therefore, the hydrodynamic evaporation of water will decrease. Also, in the presence of magnetized water, electrical conductivity as well as the moisture content of the skin increases.

In recent years, magnetized water has made a lot of progress in different fields such as industry, medicine, and agriculture (32). In a study, Hafizi *et al*. demonstrated that the mean number of corpora lutea and the height of epithelial cells in the fallopian tube were increased in pregnant female mice in the pre-implantation stage compared to the control group. Thus, magnetized water has been effective in the fertilizing of mice (33). As mentioned before, magnetized water has good effects in different fields. In the current study, SLN and NLC formulations prepared with magnetized water showed good properties. Thus, they could be used as carriers for topical drug delivery.

**Table 1:** Some changes in the properties of magnetized water which we used in this study (13)

Feature	Normal fluid	Magnetized water
The ‌‌surface tension of water (mN/m)	71	58
Dissolved oxygen in water (mg/l)	4.40 @ 70 ℃	5.73 @ 70 ℃
pH of water	7.86 @ 70 ℃	8.08 @ 70 ℃
Stability of colloidal particles in water (speed of coagulation of particles, cm^3^/s)	11 × 10^13^	9 × 10^13^
Kinematic viscosity of water (m^2^/s)	7.0387 × 10^-9^	2.0959 × 10^-12^
The density of water (g/cm^3^)	0.89646	1.00267
Number of hydrogen bonds in water	3.470	3.482

**Table 2:** Types of prepared formulation and the percentage of each component

	PP	CP	TT
Lipid phase	Precirol® 5%	Compritol® 5%	Tripalmitin 5%
Aqueous phase	Poloxamer 5% Water q.s.	Poloxamer 2.5% Water q.s.	Tween 2.5% Water q.s.

pp: Precirol®, Poloxamer; cp: Poloxamer, Compritol®; TT: Tripalmitin, Tween 80

**Table 3:** The mean diameter (z-average), PDI, and zeta potential of the various formulations

Formulation	z-average size(nm)± SD	PDI ±SD	zeta potential (mV)±SD
SLN-pp	132.4±2.1	0.220±0.02	-25.5±0.6
SLN-pp MAG	130.6±2.8	0.223±0.02	-18.1±0.9
NLC-pp	113.8±3.3	0.162±0.00	-15.9±2.1
NLC-pp MAG	101.1±2.1	0.111±0.02	-18.3±1.3
SLN-cp	161.2±2.3	0.256±0.00	-28.3±0.9
SLN-cp MAG	152.9±2.8	0.244±0.01	-29.3±1.3
NLC-cp	159.4±1.9	0.268±0.02	-19.6±1.1
NLC-cp MAG	153.5±2.3	0.133±0.01	-29.2±0.9
SLN-tt	186.0±0.5	0.177±0.01	-17.3±2.1
SLN-tt MAG	147.1±0.2	0.141±0.01	-24.4±1.2
NLC-tt	106.9±1.3	0.121±0.02	-24.3±2.0
NLC-tt MAG	101.0±1.8	0.114±0.00	-28.2±1.1

Pp : Precirol®, Poloxamer with deionized water; pp MAG :Precirol®, Poloxamer with magnetized water; cp: Compritol®, Poloxamer with deionized water; cp MAG: Compritol®, Poloxamer with magnetized water; tt: Tripalmitin,Tween 80 with deionized water; tt MAG: Tripalmitin,Tween 80 with magnetized water

A) SLN pp: Precirol®, Poloxamer with deionized water; B) SLN pp MAG: Precirol®, Poloxamer with magnetized water; C) NLC pp: Precirol®, Poloxamer with deionized water; D) NLC pp MAG:Precirol®, Poloxamer with magnetized water

D)NLC pp MAG:Precirol®, Poloxamer with magnetized water

* P <0.05, ** *P *<0.01 and *** P <0.001)

SLN: solid lipid nanoparticles; NLC:nanostructured lipid carriers

## Conclusion

The effects of magnetized water on SLN and NLC formulations were investigated in terms of particle size and moisturizing effects. All products prepared with magnetized water were more stable, smaller, and with more moisturizing effects compared with products prepared with deionized water. The results showed that in products prepared with magnetized water, the 5% SLN Precirol® had the most moisturizing effect by the *in vivo* method, and the 5% Compritol® SLN had the most moisturizing effect by the *in vitro* method. Among products prepared with deionized water, 5% Precirol® NLC and 5% Compritol® NLC had the highest moisturizing effects by *in vitro* and *in vivo* methods, respectively. Therefore, the use of magnetized water in formulations can improve the effectiveness and increase the stability of moisturizing products. 

## References

[1] Wan DC, Wong VW, Longaker MT, Yang GP, Wei F-C (2014). Moisturizing different racial skin types. J Clin Aesthet Dermatol.

[2] Derakhshanfar A, Moayedi J, Derakhshanfar G, Fard AP (2009). The role of Iranian medicinal plants in experimental surgical skin wound healing: An integrative review. Indian J Pharm Sci.

[3] Mukherjee S, Ray S, Thakur R (1998). Solid lipid nanoparticles: a modern formulation approach in drug delivery system. Eurocosmetics.

[4] Wissing SA, Müller RH (2003). Cosmetic applications for solid lipid nanoparticles (SLN). Int J Pharmaceut.

[5] Zur Mühlen A, Mehnert W (1998). Drug release and release mechanism of prednisolone loaded solid lipid nanoparticles. Pharmazie.

[6] Ahangarpour A, Oroojan AA, Khorsandi L, Kouchak M, Badavi M (2019). Antioxidant, anti-apoptotic, and protective effects of myricitrin and its solid lipid nanoparticle on streptozotocin-nicotinamide-induced diabetic nephropathy in type 2 diabetic male mice. Iran J Basic Med Sci.

[7] Wissing S, Müller R (2002). Solid lipid nanoparticles as carrier for sunscreens: in vitro release and in vivo skin penetration. J Control Release.

[8] Müller R, Dingler A (1998). The next generation after the liposomes: solid lipid nanoparticles (SLN, Lipopearls) as dermal carrier in cosmetics. Eurocosmetics.

[9] Barry B (2002). Drug delivery routes in skin: a novel approach. Adv Drug Deliv Rev.

[10] Patel D, Dasgupta S, Dey S, Roja Ramani Y, Ray S, Mazumder B (2012). Nanostructured lipid carriers (NLC)-based gel for the topical delivery of aceclofenac: preparation, characterization, and in vivo evaluation. Sci Pharm.

[11] Mehnert W, Mäder K (2012). Solid lipid nanoparticles: production, characterization and applications. Adv drug Deliv Rev.

[12] Souto E, Müller R (2008). Cosmetic features and applications of lipid nanoparticles (SLN®, NLC®). Int J Cosmet Sci.

[13] Ameri M, Aminshahidy B, Gholizadeh M (2018). Influence and application of an external variable magnetic field on the aqueous HCl solution behavior: experimental study and modelling using the taguchi method. Appl Chem Eng J.

[14] Rashidi H, Ahmadpour A, Gholizadeh M, Bamoharram FF, Moosavi F (2018). Effect of magnetized ethanol on the shape evolution of zinc oxide from nanoparticles to microrods: experimental and molecular dynamic simulation study. Adv Powder Technol.

[15] Esmaeilnezhad E, Choi HJ, Schaffie M, Gholizadeh M, Ranjbar M (2017). Characteristics and applications of magnetized water as a green technology. J Clean Prod.

[16] Golmohammadzadeh S, Mokhtari M, Jaafari MR (2012). Preparation, characterization and evaluation of moisturizing and UV protecting effects of topical solid lipid nanoparticles. Braz J Pharm Sci.

[17] Kumar VV, Chandrasekar D, Ramakrishna S, Kishan V, Rao YM, Diwan PV (2007). Development and evaluation of nitrendipine loaded solid lipid nanoparticles: influence of wax and glyceride lipids on plasma pharmacokinetics. Int J Pharm.

[18] Souto E, Wissing S, Barbosa C, Müller R (2004). Development of a controlled release formulation based on SLN and NLC for topical clotrimazole delivery. Int J Pharm.

[19] Khameneh B, Halimi V, Jaafari MR, Golmohammadzadeh S (2015). Safranal-loaded solid lipid nanoparticles: evaluation of sunscreen and moisturizing potential for topical applications. Iran J Basic Med Sci.

[20] Mosallaei N, Jaafari MR, Hanafi-Bojd MY, Golmohammadzadeh S, Malaekeh-Nikouei B (2013). Docetaxel-loaded solid lipid nanoparticles: preparation, characterization, in vitro, and in vivo evaluations. J Pharm Sci.

[21] Eshaghi Z, Gholizadeh M (2004). The effect of magnetic field on the stability of (18-crown-6) complexes with potassium ion. Talanta.

[22] Khameneh B, Iranshahy M, Ghandadi M, Ghoochi Atashbeyk D, Fazly Bazzaz BS, Iranshahi M (2015). Investigation of the antibacterial activity and efflux pump inhibitory effect of co-loaded piperine and gentamicin nanoliposomes in methicillin-resistant Staphylococcus aureus. Drug Dev Ind Pharm.

[23] Jores K, Mehnert W, Drechsler M, Bunjes H, Johann C, Mäder K (2004). Investigations on the structure of solid lipid nanoparticles (SLN) and oil-loaded solid lipid nanoparticles by photon correlation spectroscopy, field-flow fractionation and transmission electron microscopy. J Control Release.

[24] Fazly Bazzaz BS, Khameneh B, Zarei H, Golmohammadzadeh Sh (2016). Antibacterial efficacy of rifampin loaded solid lipid nanoparticles against Staphylococcus epidermidis biofilm. Microb Pathog.

[25] Wissing S, Müller R (2002). The influence of the crystallinity of lipid nanoparticles on their occlusive properties. Int J Pharm.

[26] Sator PG, Schmidt JB, Hönigsmann H (2003). Comparison of epidermal hydration and skin surface lipids in healthy individuals and in patients with atopic dermatitis. J Am Acad Dermatol.

[27] Golmohammadzadeh S, Imani F, Hosseinzadeh H, Jaafari MR (2011). Preparation, characterization and evaluation of sun protective and moisturizing effects of nanoliposomes containing safranal. Iran J Basic Medical Sci.

[28] Alimi F, Tlili M, Amor MB, Maurin G, Gabrielli C (2009). Influence of magnetic field on calcium carbonate precipitation in the presence of foreign ions. Sur Eng Appl Electrochem.

[29] Levy M, Schutze W, Fuhrer C, Benita S (1994). Characterization of diazepam submicron emulsion interface: role of oleic acid. J Microencapsul.

[30] Chibowski E, Szcześ A (2018). Magnetic water treatment–A review of the latest approaches. Chemosphere.

[31] Koshoridze S, Levin YK (2014). The influence of a magnetic field on the coagulation of nanosized colloid particles. Technical Physics Letters.

[32] Pour AN, Gholizadeh M, Housaindokht M, Moosavi F, Monhemi H (2017). A new method for preparing mono-dispersed nanoparticles using magnetized water. Applied Physics A.

[33] Hafizi L, Gholizadeh M, Karimi M, Hosseini G, Mostafavi-Toroghi H, Haddadi M (2014). Effects of magnetized water on ovary, pre-implantation stage endometrial and fallopian tube epithelial cells in mice. Iran J Reprod Med.

